# Reflections on the future of psychiatry

**DOI:** 10.1192/bji.2024.17

**Published:** 2024-08

**Authors:** Hussien Elkholy

**Affiliations:** Clinical Lecturer in Psychiatry, Department of Clinical Neuroscience, Brighton and Sussex Medical School, Brighton, UK. Email: drhelkholy@gmail.com

**Keywords:** Community psychiatry, mental health services, metacommunity, child and adolescent mental health, behavioural addictions

## Abstract

The new Deputy Editor of *BJPsych International* introduces articles in the current issue on topics as diverse as metacommunity psychiatry, child and adolescent mental health services in Australia and the Philippines, the mental health of the UK's Gypsy, Roma and Traveller populations, Indigenous mental health professionals in Bangladesh, and the relationship between spirituality and behavioural addictions.

As the new Deputy Editor of *BJPsych International*, it was a pleasure to read through the articles in this issue. In doing so, I realised it has been 7 years since publication of *The Lancet Psychiatry* and World Psychiatric Association (WPA) Commission's report on the future of psychiatry. The report addresses the opportunities and challenges that face psychiatry, covering areas including patients, societies, treatments, services, healthcare systems, mental health law, digital psychiatry and the training of future psychiatrists.^1,2^

## Evolving services and systems

Focusing on services and healthcare systems, there is no doubt that psychiatry is one of the fastest evolving and adapting medical specialties. The impact of the COVID-19 pandemic is a good example of how practice, training and teaching evolved to meet individual and societal needs. Another example is the move from institutionalisation to community psychiatry. In this issue of *BJPsych International*, an interesting and thought-provoking article titled ‘Metacommunity: the current status of psychiatry and mental healthcare and implications for the future’ discusses the history and origins of community psychiatry and challenges facing it.^3^

While still on the topic of services, it is crucial to stop and reflect on the status and needs of current systems. I invite you to read two articles in this issue shedding light on child and adolescent mental health systems in Australia and Philippines. Both articles provide insight on their respective mental health services and challenges they face.^4,5^

## Culturally informed psychiatry

The WPA–*Lancet Psychiatry* Commission on the future of psychiatry^1^ also highlighted the role of psychiatrists in societies and the importance of considering varying needs in diverse populations. This includes advocacy for the rights of people living with mental illness and against discrimination, working with people with lived experience, families, carers and communities. In this domain, this issue has two interesting articles. The first addresses the situation of Gypsy, Roma and Traveller (GRT) populations in the UK. The authors argue the need for a culturally informed approach to develop services accessible to GRT communities, who have considerably worse mental health outcomes than the general population.^6^

The second article looks at the situation from a different angle: the practitioners. The authors discuss the need for having more Indigenous mental health practitioners. They argue that Indigenous people are less likely to seek healthcare, owing to the insufficient number of healthcare professionals representing their communities. Focusing on Bangladesh, they hope that increasing representation among mental health professionals will ensure that mental health services in Bangladesh are inclusive and embracing the country's diversity.^7^

## Spirituality and of behavioural addictions

The past few years have seen an increased interest in behavioural addiction. The inclusion of gaming disorder in classificatory systems has brought more attention to the topic and the variety of behaviours that can be considered problematic. Kanabar et al^8^ discuss in their narrative review the complex relationship between spirituality and different forms of behavioural addiction. The authors concluded with the potential value of understanding this relationship in enhancing the care provided, and the need for more robust research on the topic.

## Data Availability

Data availability is not applicable to this article as no new data were created or analysed in this study.
